# OmniGA: Optimized Omnivariate Decision Trees for Generalizable Classification Models

**DOI:** 10.1038/s41598-017-04281-9

**Published:** 2017-06-20

**Authors:** Arturo Magana-Mora, Vladimir B. Bajic

**Affiliations:** King Abdullah University of Science and Technology (KAUST), Computational Bioscience Research Center, Thuwal, 23955-6900 Saudi Arabia

## Abstract

Classification problems from different domains vary in complexity, size, and imbalance of the number of samples from different classes. Although several classification models have been proposed, selecting the right model and parameters for a given classification task to achieve good performance is not trivial. Therefore, there is a constant interest in developing novel robust and efficient models suitable for a great variety of data. Here, we propose OmniGA, a framework for the optimization of omnivariate decision trees based on a parallel genetic algorithm, coupled with deep learning structure and ensemble learning methods. The performance of the OmniGA framework is evaluated on 12 different datasets taken mainly from biomedical problems and compared with the results obtained by several robust and commonly used machine-learning models with optimized parameters. The results show that OmniGA systematically outperformed these models for all the considered datasets, reducing the F_1_ score error in the range from 100% to 2.25%, compared to the best performing model. This demonstrates that OmniGA produces robust models with improved performance. OmniGA code and datasets are available at www.cbrc.kaust.edu.sa/omniga/.

## Introduction

**Figure 1:**
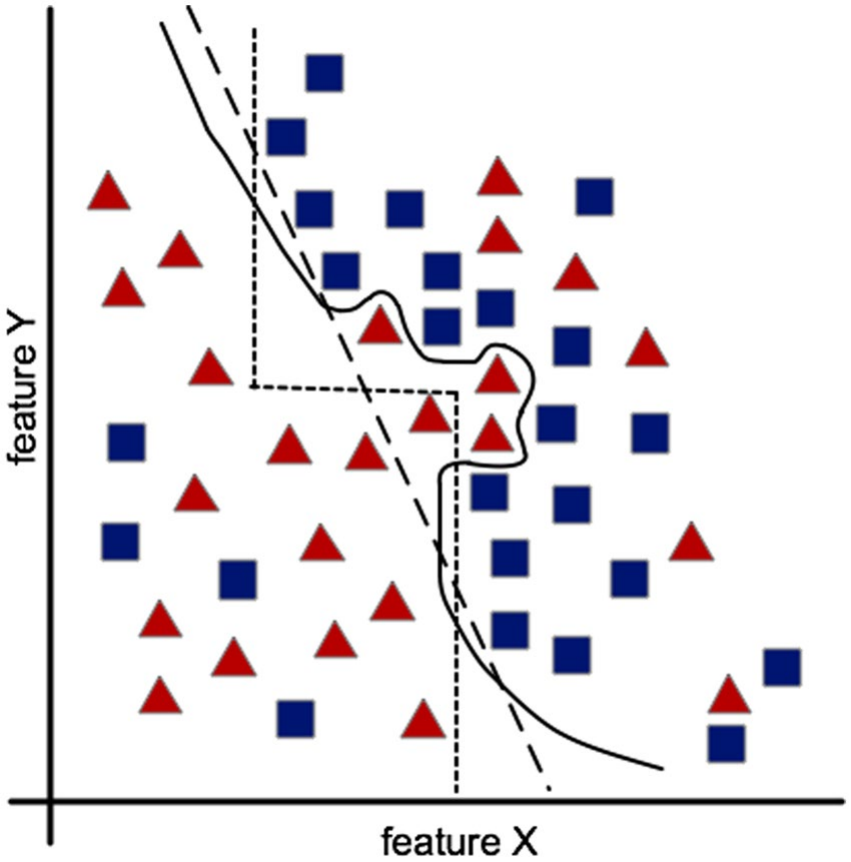
DT classification boundaries. Example of univariate (dotted line, three rules), linear multivariate (dashed line, one rule) and nonlinear multivariate (solid line, one model) DTs.

Problems from a wide range of domains may be formulated as classification tasks including those in biology and chemistry, i.e., disease classification^1–5^, drug toxicity and response^6, 7^, and many others^8–10^. However, the performance of a classification model depends on its parameters and the nature of the data. Therefore, a particular classification model may work better for a given dataset than other models^11, 12^. Consequently, selecting an optimized model and its parameters for each dataset is crucial. Although there are several classifier families (statistical, ensemble methods, rule-based, etc.), we start by defining decision trees (DTs) and their evolution for creating more robust models, which combines linear and non-linear models in the DT nodes. DTs are popular machine-learning models for pattern recognition and classification, and several algorithms have been proposed for the induction of DT classifiers from data^13^. A DT implements a set of classification rules inferred from a labeled dataset of samples. Each non-terminal node implements a locally optimal discriminant function, which splits the data into two or more partitions until all samples in the node belong to one class or until a stopping criterion is met. DTs may be broadly classified into three groups based on the type of discriminant function used at each non-terminal node: (1) univariate DT, where each non-internal node uses one feature to split the data, (2) linear multivariate DT, where the discriminant function consists of a linear combination of the features, and (3) nonlinear multivariate DT, where each non-terminal node uses nonlinear function of data features to split the data (Fig. 1).

While univariate DTs offer several advantages, i.e., scalability, simplicity and understandability^14, 15^, their performance may be compromised when dealing with difficult pattern recognition problems that may require extremely complex DTs. Consequently, several DT induction algorithms have been proposed with the aim of producing models that can perform better in both simple and complex classification tasks. Linear multivariate CART induction algorithm^16^ uses an iterative back fitting algorithm based on a deterministic hill-climbing technique to individually optimize coefficients for the linear combination of features at each node. Oblique classifier 1^17^ (OC1) was later developed as an extension of CART to avoid local minima during the coefficient optimization phase. Linear multivariate DTs were modified to support classification problems with more than two classes^18, 19^. Although other DT induction algorithms have been proposed (FACT^20^, QUEST^21^, and CRUISE^22^), the prediction performance is not statistically different from results obtained by CART or univariate C4.5 DTs^23^. The studies mentioned above have shown that linear multivariate trees generally produced smaller DTs (although with more complex nodes) with competitive and in some cases better accuracy than univariate DTs.

At the expense of simplicity and model interpretation, nonlinear multivariate DTs define a hypersurface with an arbitrary shape that, in principle, may separate the data more accurately, particularly for difficult classification problems^24–26^. Guo and Gelfand^26^ proposed to use small artificial neural networks (ANNs) at each non-terminal node to model class separation. Authors concluded that the method generally produced lower error rates compared to CART and similar to those obtained by ANNs. Nevertheless, the use of ANNs in each non-terminal node increased the number of parameters that had to be defined, i.e., the number of hidden layers, the number of nodes in the layers, learning rate, etc. To avoid this, Sankar and Mammone^27^ proposed the neural tree networks (NTN) method, which uses a single layer perceptron or neuron with a sigmoid activation function in each non-terminal node. NTNs are expanded using a heuristic learning algorithm based on the minimization of the absolute error (L1 norm) of the neurons. NTN method was able to achieve a similar prediction accuracy compared to the best ANN model but can be achieved without the hassle of determining an optimal ANN structure for a particular problem. Yildiz and Alpaydin^28^ proposed to use Fisher’s linear discriminant analysis in each non-terminal node to generate binary trees (LDA DT). They compared results from their method against other DT induction algorithms (univariate ID3, linear multivariate CART, linear NTN and LMDT) and concluded that univariate ID3 achieves the best accuracy when the data are small and features are not correlated. However, LDA DT achieved better accuracy than ID3 DT in more complex problems. Their method outperformed the results from linear multivariate CART and produced the same accuracy as linear NTN. Despite the improvements achieved by LDA DT, the method still presents the limitations of linear classifiers, which results in lower performance when dealing with complex classification problems. As a consequence, Kumar and Rani^29^ proposed the use of the direct fractional-step linear discriminant method, in which each node uses a DF-LDA to form a nonlinear DT model. However, studies^26, 28^ have shown that complex models, i.e., multivariate trees, increase the risk of overfitting the data for problems where data are simple (features are not correlated) or where data are scarce (reason why ID3 performed better than LDA DT in some cases). To avoid these problems and still make use of the advantages of nonlinear models, Yildiz and Alpaydin^30^ proposed the Omnivariate DTs (ODTs). ODT induction algorithms allow the tree to use any of the above-mentioned discriminant functions (i.e., univariate, linear multivariate or nonlinear multivariate) in non-terminal nodes. The original ODT induction algorithm uses comparative statistical tests of accuracy to dynamically select the most appropriate discriminant model at a node with the sub-problem defined by the data reaching the node. The authors also suggested that model selection plays a crucial role in the induction of ODTs. Therefore, several studies^25, 31–33^ developed new strategies for model selection in the nodes, considering more robust or faster statistical measures. Yildiz and Alpaydin^30–32^ observed that ODTs were never outperformed by DTs with all nodes from the same class (univariate, linear multivariate or nonlinear multivariate). However, the use of different discriminant functions in the nodes and the use of more complex models increase the intricacy of DT generation and DT pruning strategies. Moreover, using nonlinear models at each non-terminal node, i.e., ANNs, introduce the challenges of parameter tuning of that particular model. For the purpose of simplifying this task, previous studies^26, 30^ used small and fixed ANN structures, which may result in suboptimal local classification models. Moreover, ODTs, as well as other DT induction algorithms (ID3, C4.5, CART, etc.), implement local operations to find the feature (or model in the case of ODTs) and the split threshold values that best splits the data in a particular non-terminal node. These local partitioning heuristics may produce suboptimal solutions, as they do not have the information of the data and the model as a whole.

In this study, we propose OmniGA, a generic framework that uses a parallel genetic algorithm (GA) for the optimization of ODTs. In the OmniGA framework, the GA encodes the structure of the tree and the parameters for each model in non-terminal nodes. The GA simultaneously optimizes the following processes:

1. Model selection: GA attempts to find the best model type for each non-terminal node (linear or nonlinear model).
2. ODT model simplification: GA prunes non-terminal nodes.
3. Model optimization in non-terminal nodes: GA finds the set of optimized parameters for models used at each non-terminal node.
4. Threshold optimization: GA attempts to find an optimal threshold value that best discriminates data classes at each non-terminal node.

Moreover, OmniGA takes advantage of the hierarchical model of the DT to construct a deep learning (DL) structure, where each successive non-terminal node uses as new features the predicted probabilities for each sample in the parent node. Finally, we describe and evaluate an ensemble model^34^ that uses a set of OmniGA models encoded into the GA strings to produce the final prediction. This ensemble method takes advantage of the different models produced by the GA while decreases model variance^35^.

Usually, new proposed classification models are only compared against other models within the same classifier family. However, the goal of our study is to develop a robust and generalizable model able to outperform existing commonly used classification methods. For this, we measured OmniGA performance on 12 different classification problems mainly from biomedical domain and compared it against four other classification methods with their optimized parameters, i.e., ANNs (as described in^34^), random forest^36^ (RF), multinomial logistic regression^37^ (MLR) and C4.5 DTs^38^. The results from these comparisons convincingly demonstrate that OmniGA performance represents an improvement compared to that of the other models we tested and that it was never outperformed by these other models.

## Results

The key contribution of our study is the development of OmniGA, a new robust framework for classification tasks capable of learning from a great variety of data (despite data complexity, the number of samples, imbalances, etc.). OmniGA combines several machine-learning techniques such as GAs, DL, synthetic minority oversampling technique^39^ (SMOTE), early stopping of model training (ES), ensemble learning, and different types of classifiers for deriving robust ODTs. The technical details of OmniGA are presented in the Methods section.

To evaluate the utility of the OmniGA framework, we used 12 different datasets (see Table 1) and compared the results to those obtained by other robust and commonly used classification models, i.e., ANN, RF, MLR and C4.5 DT. To ensure a fair comparison with the baseline reference models, we performed a grid search of the parameters for each model and dataset (Supplementary Table S1 shows the parameter values considered for the grid search and Supplementary Table S2 shows the final selected parameters that achieved the best performance on the validation set). Specifically, models tuned are MLR (ridge parameter), RF (number of trees and number of random features), ANN (learning rate and number of nodes in the hidden layer) and C4.5 DT (confidence factor and minimum number of instances for further tree expansion). In this way, we obtained optimized reference models. OmniGA framework also performs a grid search for the crossover and mutation probabilities. Each of these combinations produces different models. From the resulting models, the one with the highest performance on the validation set is selected as the final model. Note that in OmniGA algorithm, the GA optimizes the ODT structure and performs the tuning of the models in the non-terminal nodes. Although new hybrid classification algorithms have been proposed (LibD3C^40^, DTNB^41^, among others^42, 43^), there are several justifications for the selection of the reference classification models used in this study. An extensive empirical study^11^ has shown that RF produced the best average performance over the UCI machine-learning repository database and therefore suggest it should be considered as the reference gold standard. Moreover, RF have shown to produce outstanding results in some bioinformatics applications^44–46^. In addition, ANNs achieved the best average performance on two-class classification problems^11^. Finally, we also considered MLR and C4.5 as they are consistently used to compare DT induction algorithms^23^.

**Table 1:** Datasets description.

Dataset	# Samples	# Features	Class ratio
TF combinations (TFC)	2,418	3,186	1:1.04
Lung tissue (LT)	2,387	351	1:10
Esophagus tissue (ET)	1,474	351	1:10
TIS prediction (TIS)	24,832	47	1:1
EEG eye state (EES)	14,980	14	1:1.22
Heart statlog (HS)	270	13	1.25:1
Pima Indian diabetes (PID)	768	8	1:1.86
Central nervous system (CNS)	60	7,129	1:1.85
Colon tumor (CT)	62	2,000	1:1.81
Synthetic (SYN)	2,000	65	1:1
German credit (GC)	1,000	24	2.33:1
Credit card clients (CCC)	30,000	24	1:3.52

Predictive results are normally reported in terms of accuracy. However, this measure is not well suited for imbalanced datasets. For instance, consider a dataset with 90% of the observations from class 1 and the rest from class 2; a model that only learns class 1 samples would report ~90% accuracy. Therefore, in order to account for imbalanced datasets, we used the *F*_*1*_ score (we will refer to it as *F*_*1*_, hereafter), which accounts for both precision and sensitivity (Supplementary Table S3). However, we also provide results in terms of other performance measures (Supplementary Table S4 shows the accuracy, sensitivity, specificity and precision measures for each dataset). Moreover, all reported results are obtained using 2/3 of the data for training and the remaining for testing (Supplementary Table S5 shows the results using a 3-fold cross-validation technique).

**Figure 2:**
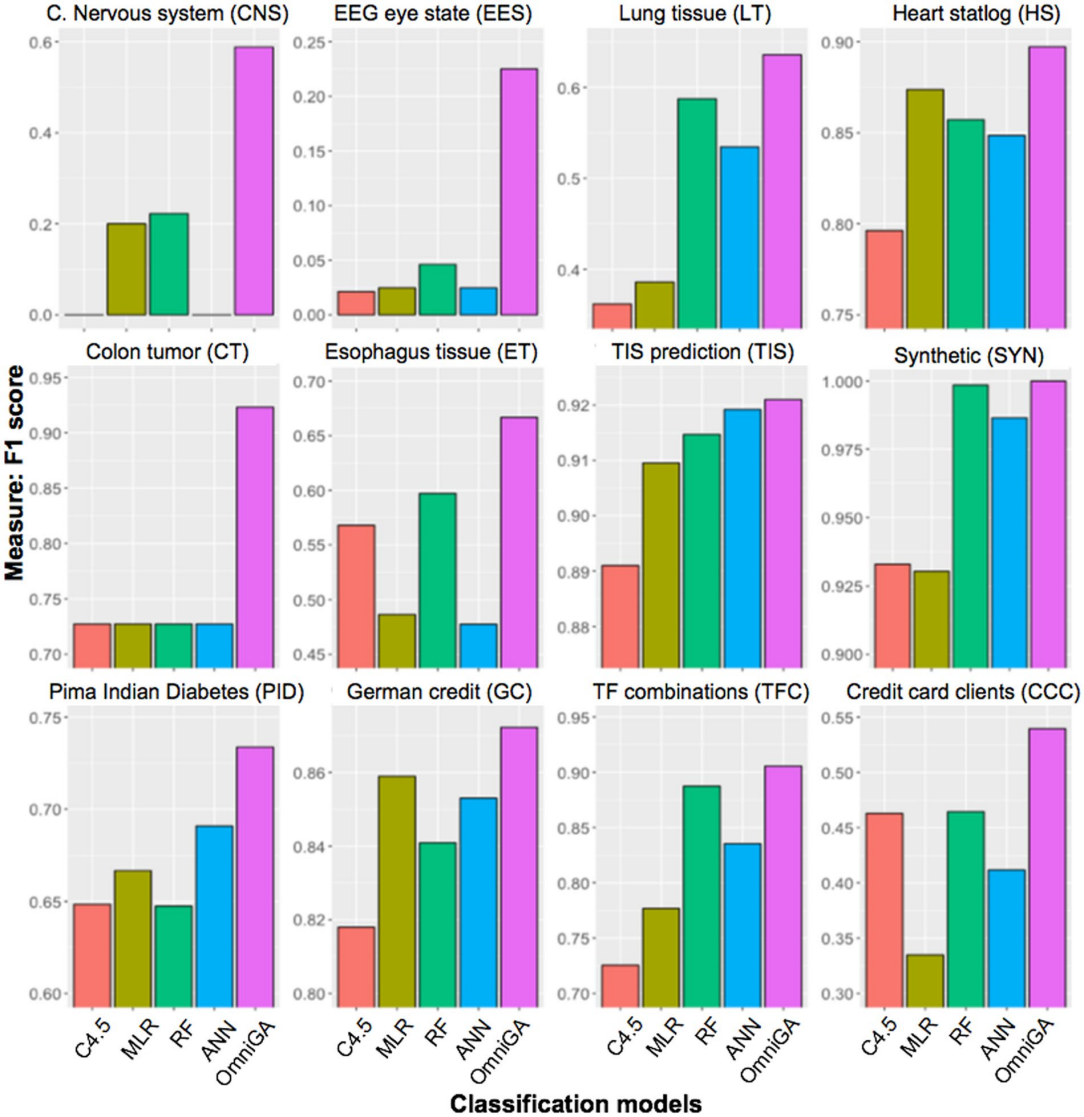
Models performance. *F*_*1*_ for each model on the twelve considered datasets.

Figure 2 shows the *F*_*1*_ values for each model in the respective dataset. We observe that from the existing models (ANN, RF, MLR and C4.5) there is no model that consistently achieves the best performance. For instance, MLR (yellow bar) outperformed all existing models in two datasets (HS and GC), while ANN (blue bar) outperformed other models in two datasets (TIS and PID), and finally, RF (green bar) achieved best results on the eight remaining datasets. Moreover, we observe the inability of both C4.5 and ANN to learn the positive class (minority class) in CNS and EES datasets as these models achieve *F*_*1*_ of 0% and 0.2%, respectively.

Results show that the OmniGA framework (purple bar) was never outperformed by any of the other tested models (OmniGA parameters are listed in Supplementary Table S6). Moreover, OmniGA improved results in all of the 12 considered datasets, showing that OmniGA consistently produced robust models despite the complexity and dataset size.

Table 2 compares OmniGA results against those obtained by the best performing model for each dataset. Here we define relative *F*_*1*_ error, *F*_*1*_^*relative_error*^, as

1 $${{F}_{1}}^{relative\_error}({m}_{1},{m}_{2})=\frac{{{F}_{1}}^{error}({m}_{1})-{{F}_{1}}^{error}({m}_{2})}{{{F}_{1}}^{error}({m}_{1})}$$

where

2 $${{F}_{1}}^{error}(m)=1-{F}_{1}(m)$$

and where m_i_ stands for the classification model. From these results, we observe the considerable reduction of relative *F*_*1*_ error e.g. for SYN, CT, CNS, ESS and HS datasets compared to the best performing model tested as 100%, 71.8%, 47%, 18.7% and 18.5%, respectively.

**Table 2:** Classification error reduction. Comparison between best performing model and OmniGA framework.

Dataset	Best performing existing model	Reduction of the *F*_*1*_^*relative_error*^ (%)
TF combinations (TFC)	RF	16.06
Lung tissue (LT)	RF	11.71
Esophagus tissue (ET)	RF	17.28
TIS prediction (TIS)	ANN	2.25
EEG eye state (EES)	RF	18.75
Heart statlog (HS)	MLR	18.54
Pima Indian diabetes (PID)	ANN	13.83
Central Nervous system (CNS)	RF	47.05
Colon tumor (CT)	C4.5, MLR, RF, ANN	71.79
Synthetic (SYN)	RF	100.00
German credit (GC)	MLR	13.06
Credit card clients (CCC)	RF	14.03

*Best performing existing model* refers to the model (MLR, ANN, RF and C4.5) achieving the best *F*_*1*_ performance in a given dataset. *Reduction of the F*_*1*_^*relative error*^ denotes the relative error reduction of OmniGA with respect to the best performing model as defined in Equation 1.

## Discussion

The performance of classification models depends on model parameters and the nature of the problem. Therefore, choosing the right model for a given problem is crucial. In search of a robust and generalizable model, we developed OmniGA, a novel framework to optimize the structure of ODTs. OmniGA uses a parallel GA to optimize: (1) a split threshold for splitting the data, (2) model selection for each non-terminal node, (3) parameters for models in non-terminal nodes, and (4) pruning of the tree. OmniGA takes advantage of the hierarchical structure of the tree and implements a DL structure in some ODT structures depending on the data. DL origins date to the mid 80’s when Rumelhart *et al*.^47^ described the back-propagation algorithm for ANNs. DL algorithms consist of a multi-layered architecture that transforms the data representation at one level to a higher and more abstract level^48^ (where features are learned from the data itself). In an OmniGA model, it is possible to have multiple ANNs or MLRs or a combination of both in a decision branch of the OmniGA, which would constitute a DL structure. Additionally, in OmniGA, the outputs of the parent non-terminal node (prediction scores for each sample and split threshold derived from the parent non-terminal node) are used as new features for samples reaching the child non-terminal node. For instance, consider sample *s* = [*s*_*1*_, *s*_*2*_, *s*_*3*_, …, *s*_*d*_] where *s*_*j*_ represents a feature and *d* denotes the number of features. A non-terminal node of the ODT would assign a prediction score *s*_*p*_ and the split threshold *s*_*t*_ for each sample *s*. These two numerical values represent an abstraction of the feature vector and constitute the novel features learned from the original data reaching the parent non-terminal node. These two values, s_t_ and s_p_, are later added as two additional features to the original feature vector for the sample, resulting in *s* = [*s*_*1*_, *s*_*2*_, *s*_*3*_, …, *s*_*d*_, *s*_*p*_, *s*_*t*_]. Thus the combination of the above characteristics conforms the DL structures in an OmniGA. Moreover, a subset of the resulting GA strings is used to build an ensemble model and therefore reduce the prediction variance of a single model. Finally, we defined different add-ons, the use of which results in different ODT induction strategies, i.e., using SMOTE, ES and DL at each non-terminal node. The rationale behind using SMOTE is that since ODTs recursively split the data based on the samples predicted probabilities, it is often the case that non-terminal nodes receive an imbalanced partition of the data with respect to the classes. Since data imbalances may have a crucial effect on the performance of classification models^49^, this is, therefore, an important factor to consider in model training in non-terminal nodes of the ODTs. Finally, the ES add-on consists of prematurely stopping the model training in non-terminal nodes. Clearly, complex and fully trained models in non-terminal nodes may possibly result in smaller ODTs. However, the use of semi-trained and less complex models may partition the data in more subsets and hence increase the robustness of the model.

The process of using the set of different OmniGA models with different add-on configuration could be regarded as a GA with parallel populations (where each subpopulation evolves differently) without individual migration. Supplementary Table S7 shows the best performing add-on configurations for each dataset (chosen based on the performance on the validation set).

We compared OmniGA framework performance against other robust and commonly used classification models, namely ANN, RF, MLR and C4.5. We used 11 datasets from different domains and one consisting of synthetic rules to compare our framework against the four above-mentioned models (C4.5, MLR, ANN and RF) with their optimized parameters for each of the datasets. Results demonstrate that the OmniGA framework improved results in all of the 12 datasets from Table 1, showing that OmniGA consistently produced robust models despite the complexity, size of the data or number of features used to describe the data.

The OmniGA framework considerably reduced *F*_*1*_^*error*^ compared to the best performing model tested. Notably, the best improvements correspond to the highly imbalanced and scarce datasets CT and CNS containing 62 and 60 samples, respectively (reduction of the relative *F*_*1*_ error by 71.8% and 47%, respectively). These results demonstrate the capability of our framework to produce robust and generalizable models even when the data are limited and scarce.

## Methods

In this section, we first describe the datasets considered to assess the performance of our framework and the data processing, followed by the GA configuration and the encoding technique. Finally, we discuss in detail the structure of the OmniGA framework.

### Datasets

In order to assess the performance of our OmniGA framework, we selected 12 datasets with different characteristics, i.e., datasets with a significant imbalance in the number of samples with respect to the classes, limited in the number of samples, as well as described by a large number of features. The first four datasets were obtained from recent studies^50–53^, five datasets were taken from the UCI machine-learning repository^54–57^, two from the Kent Ridge biomedical datasets^58–60^ and the last dataset consists of a set of synthetically generated rules (using DatGen www.datasetgenerator.com). The characteristics of these datasets are described in Table 1 (See Supplementary Material 1 for dataset details).

### Data processing and feature selection

Feature values for all datasets were normalized to have values within the range of [−1, 1]. Feature selection was performed only on datasets described by more than 300 features. We used the gain ratio method from WEKA v3.6.12^61^ to evaluate and rank the features. We retained only the top 300 ranked features for further analyses (see Supplementary Material 2).

### Genetic algorithm (GA) design

#### Encoding models in non-terminal nodes

**Figure 3:**
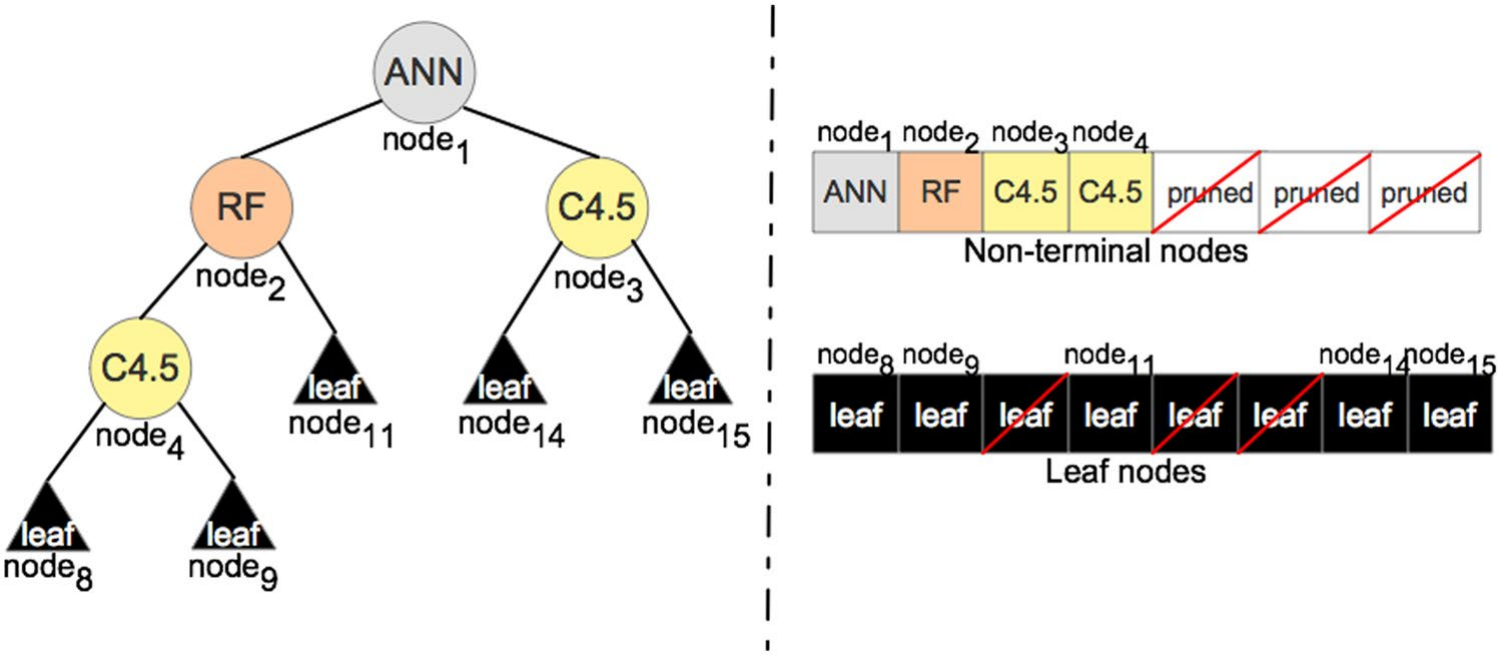
Schematic representation of an ODT model. ODT model is encoded by the GA string in a breadth-first order.

OmniGA encodes the parameters of four different models, i.e., ANN, RF, MLR and C4.5 from WEKA v3.6.12. Nodes are encoded into the GA string following the breadth-first search order. Figure 3 illustrates an example of a resulting ODT where non-terminal nodes 5, 6 and 7 were pruned from the tree. We developed two variants of GA encoding: the first only encodes the parameters for models in non-terminal nodes, while the second encodes the model parameters as well as the early stopping (ES) of training for the models (we refer to this as ‘ES add-on’). We describe in detail the encoding ODT into a GA string in Supplementary Material 3.

#### The objective function and GA operators

The fitness value for each GA string in the population consists on the statistical performance measure. In our tool (www.cbrc.kaust.edu.sa/omniga/), the measure may be, for example, accuracy, precision or *F*_*1*_ (see Supplementary Table S3).

**Figure 4:**
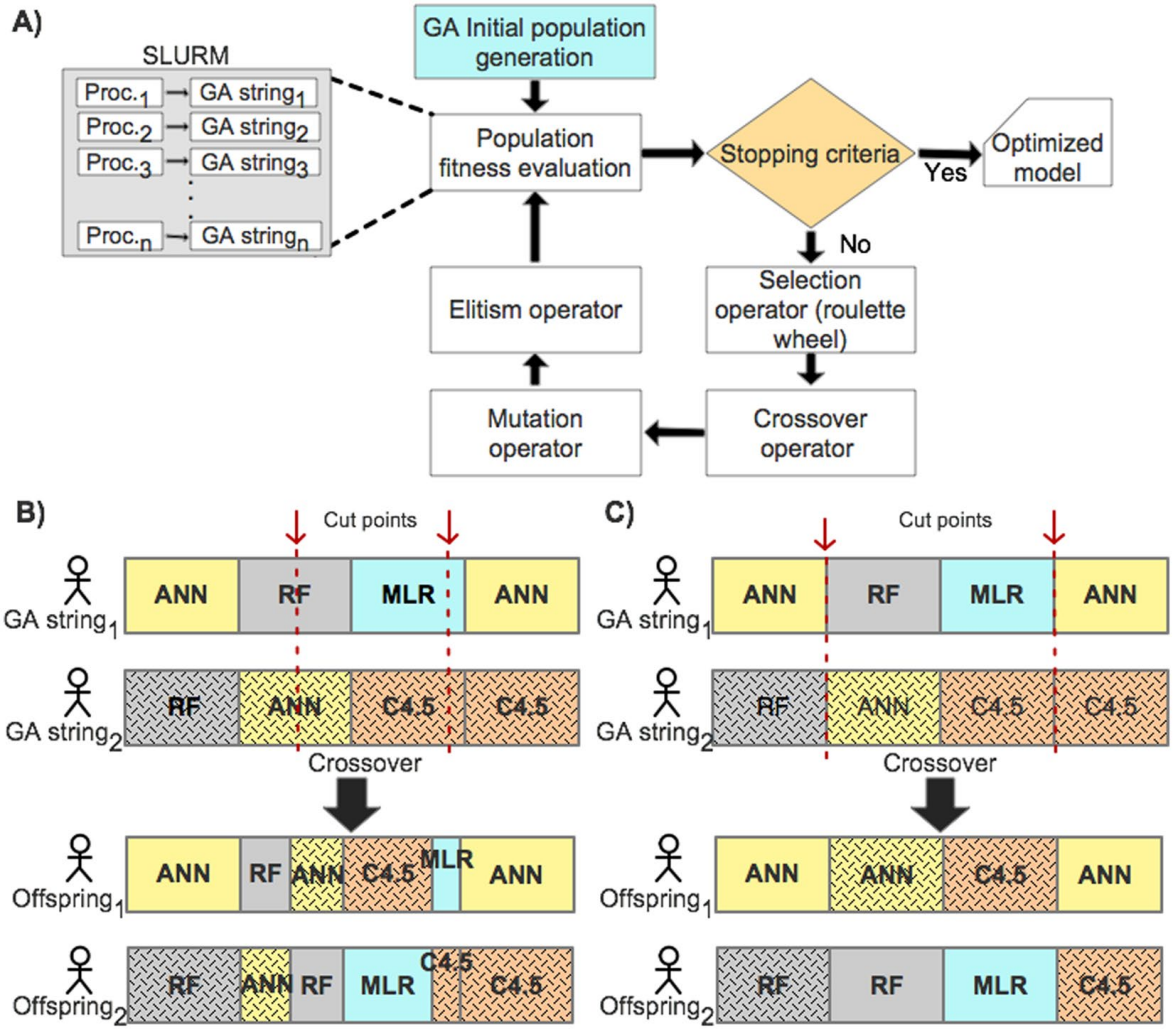
(**A**) Schematic representation of the GA execution. (**B**) Crossover inconsistencies when considering random cut points at an arbitrary bit. (**C**) Consistent crossover scheme, where cut values must be either at the beginning or end of an encoded model.

The fitness value is calculated from the validation set (15% exclusively reserved from the training data). Evaluation of the fitness values for the entire GA population is implemented as a parallel execution using the SLURM system^62^, where the evaluation of each GA string is computed by an independent process (Fig. 4A). Supplementary Table S8 shows the running time of the OmniGA framework. The fitness values for each GA string in the population are scaled by subtracting the fitness value of the worst GA string in the population, i.e., $new\,Fitnes{s}_{i}=old\,Fitnes{s}_{i}-worst\,Fitness$. This procedure intends to eliminate those GA strings that are least likely to converge to a good solution. We used the ‘roulette wheel’ fitness-based selection operator^63^, to select parents for the crossover operator.

In OmniGA, the crossover operator selects two random cut points from both parents (generating three splices from the GA string). However, GA strings encode different models (and their parameters), hence random cut points at an arbitrary bit are likely to happen within the bits encoding the model parameters and hence generating inconsistent offspring which may confuse the GA search (Fig. 4B). Consequently, we defined cut values as the *i*-th node in the GA string (Fig. 4C). The resulting offspring replace the old population, i.e., the population evolves whilst keeping the same number of strings. Moreover, to ensure that the best solution remains in the population, OmniGA uses an elitism operator. Such an operator replaces the worst fitted string in the current iteration by the fittest GA string from the previous iteration.

Finally, we implemented three different mutation strategies depending on the bit where a mutation occurs: (1) mutation in the bit encoding the model type, changes the bit to a random integer value within range of [−1, 3], 2) mutation in the threshold bit generates a random numeric value within range of [−0.65, 0.65], and 3) mutation at any other binary bit simply inverts the current value (0 → 1 or 1 → 0).

### OmniGA methods

In this subsection, we first describe the methods for the induction of ODTs. The set of optimized ODTs (generated by the GA) forms an OmniGA model. Finally, we describe the different add-ons for an OmniGA model induction, which as an ensemble constitute the OmniGA framework.

#### ODT model

ODTs use linear or nonlinear models to split the data in each non-terminal node. Each of the models produces two outputs corresponding to the probabilities for a sample to belong to class 1 or class 2, respectively. Algorithm 1 shows the pseudo code for the partitioning of the data based on the predicted probabilities, where the threshold is the variable encoded in the GA string for a given non-terminal node.

**Table:** 

**Algorithm 1** Data splitting algorithm
**for** each sample S in data
**if** PredictionScore_C1_ ≥ | PredictionScore_C2_
**if** PredictionScore_C1_ ≥ GA.threshold
S → Class 1
**else**
S → Class 2
**else**
**if** PredictionScore_C2_ ≤ GA.threshold
S → Class 2
**else**
S → Class 1

An ODT recursively expands until one of the following three stopping criteria is met:

- Maximum ODT depth: recursive ODT expansion stops if a maximum depth of 5 is reached (max. 31 nodes).
- Node impurity: ODT recursion stops if the node contains samples from only one class (clean node) or if the ratio of samples from the two classes is less or equal than 1/500.
- The minimum number of instances: this case considers only the number of samples in a node independently of their class labels. To account for small and large datasets, we set the minimum number of instances to 100 for datasets containing more than 2,000 training samples or 5% of training samples for datasets with less than 2,000 samples.

**Figure 5:**
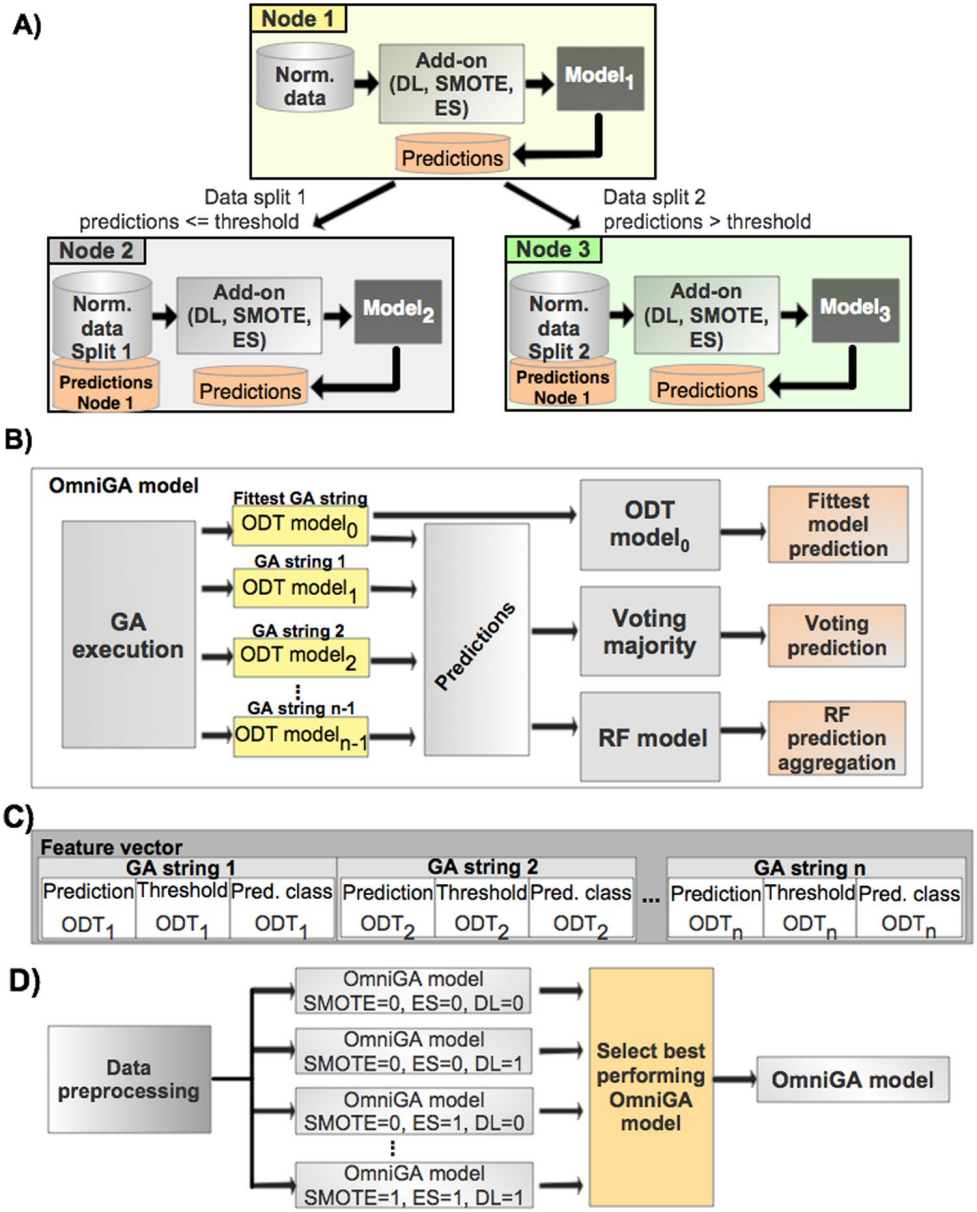
Illustration of the ODT structure and the OmniGA framework. (**A**) Schematic representation of the method followed for each non-terminal node in the ODT. (**B**) Induction process for an OmniGA model. (**C**) Illustration of the feature vector used for the deep learning module in the OmniGA model. (**D**) OmniGA framework consisting of eight OmniGA models using different add-on configurations.

Each non-terminal node in an ODT contains an add-on module, which allows for data transformation before model training or for training early stopping, as shown in Fig. 5A.

The three add-on capabilities are:

- Early stopping (ES) for model training in non-terminal nodes: as described in the GA methods, a GA string may encode a parameter to prematurely stop the training of a model in non-terminal nodes.
- Data imbalance reduction: we used the synthetic minority oversampling technique^39^ (SMOTE) to reduce data imbalances in data partitions at each non-terminal node. The synthetic samples are local to a particular node and are not inherited by the child nodes. SMOTE is performed by using WEKA v3.6.12 and was only applied when the data imbalance exceeded a ratio of 1:5. In such cases, data imbalance was reduced to a 1:2 ratio by increasing the minority class by a percentage given by
  3 $$percentage\,increase=(\frac{majority}{minority}-1)\times 50$$
  where *minority* and *majority* correspond to the number of samples in the minority and majority classes.
- Deep learning structure (DL): the prediction probabilities for each sample and split threshold are forwarded from the parent node to the child nodes. These probabilities and threshold are concatenated to the original set of features for each sample. Considering Fig. 5A, the split threshold, and probabilities generated for samples in node 1 are forwarded to node 2 and node 3 and are concatenated to the original feature set for each sample accordingly.

#### OmniGA model

An OmniGA model uses a GA to find competitive ODT models. Each OmniGA model produces three predictions for a sample (Fig. 5B):

- First prediction: prediction based on a single ODT model encoded into the fittest GA string.
- Second prediction: we constructed an ensemble method by considering a set of GA strings to perform voting majority. There are in total *m* evaluations of different ensembles with different numbers of GA strings, where *m* is equal to $GA\,population\,size-3$. As such, the first ensemble includes the fittest GA string + 2 randomly chosen GA strings (ensemble of three models), the second ensemble includes the fittest GA + 3 randomly chosen GA strings (ensemble of four models), and so on. At the end, the ensemble that produced the best discrimination based on the validation set is selected for the final majority voting ensemble model.
- Third prediction: we implemented a module that considers the prediction probabilities (instead of only the predicted class) as input for training an RF model. The number of random trees in an RF model is experimentally found in order to increase the performance as observed on the validation set. In this module, each sample is defined by a feature vector of size equal to 3 × *GA population size*. The three features for each GA string in the population are defined as follows: (**a**) predicted scores within range of [−1, 1] where positive and negative values indicate class 1 and class 2, respectively, (**b**) split threshold used in the ODT model for that particular sample at the non-terminal node previous to the leaf node, and (**c**) the predicted class label (Fig. 5C).

The prediction scheme considered for the final model is chosen by evaluating the performance of the OmniGA model for the three predictions independently based on the validation set. A comparison of the three prediction schemes is given in Supplementary Table S9.

#### OmniGA framework

The OmniGA framework consists of a set of eight OmniGA models (computed in parallel) with different add-on parameters (Fig. 5D). The eight different induction configurations are the result of combining the three add-ons described in the ODT model induction ($\#combinations={2}^{3}$), i.e., first the OmniGA model is trained without any of these add-ons (SMOTE = 0, ES = 0, DL = 0), the second model considers only the DL structure (SMOTE = 0, ES = 0, DL = 1), etc. The final output is the best performing OmniGA model for a given problem based on the performance observed on the validation set.

## Electronic supplementary material

Supplementary material
