# Supplementary material for: OmniGA: Optimized Omnivariate Decision Trees for Generalizable Classification Models
Source: Sci Rep. 2017 Jun 20;7:3898. doi: 10.1038/s41598-017-04281-9 (PMC5478657; doi:10.1038/s41598-017-04281-9)
Supplement: Supplementary file 1 — Supplementary material [file 41598_2017_4281_MOESM1_ESM.pdf]

Supplementary Material

OmniGA: Optimized Omnivariate Decision Trees for Generalizable Classification Models

Arturo Magana-Mora[1] and Vladimir B. Bajic[1,*]

King Abdullah University of Science and Technology (KAUST), Computational Bioscience Research Center, Computer, Electrical and Mathematical Sciences and Engineering Division, Thuwal, 23955-6900, Saudi Arabia

* Corresponding author: vladimir.bajic@kaust.edu.sa

Supplementary Tables

Supplementary Table S1: Parameters grid search

	Model	Min	Max	Increment
**OmniGA**	Crossover prob.	0.55	0.95	0.05
	Mutation prob.	0.05	0.25	0.05
**MLR**	Ridge	2^{-30}	2^{-1}	Exponential from -30 to -1
**C4.5**	Confidence factor	2^{-25}	$2^{-0.5}$	Exponential from -25 to -0.5
	# min inst	2	52	10
**RF**	# trees	10	210	20
	# features	5	# features in data	25
**ANN**	# nodes	5	50	5
	Learning rate	2^{-9}	2^{0}	Exponential from 0 to -9

Supplementary Table S2: Optimal parameters for single models.

**Dataset**	ANN		RF		C4.5		MLR
	# nodes	Learning rate	# trees	# features	Conf. factor	# min. inst.	Ridge param.
**TF combinations (TFC)**	5	0.125	190	55	3.54E-01	42	4.8828E-04
**Lung tissue (LT)**	10	0.015625	30	80	0.044194	22	0.5
**Esophagus tissue (ET)**	25	0.0039062	70	80	0.70711	32	0.5
**TIS prediction (TIS)**	40	0.0078125	90	5	0.35355	42	0.03125
**EEG eye state (EES)**	30	0.008227	30	5	0.022097	2	0.5
**Heart statlog (HS)**	25	1	170	5	0.022097	2	0.125
**P. Indian diabetes (PID)**	5	0.25	30	5	0.17678	2	0.125
**C. nervous system (CNS)**	5	0.0019531	50	5	0.70711	22	0.5
**Colon tumor (CT)**	5	1	10	55	0.70711	12	1.9073E-06
**Synthetic (SYN)**	15	0.5	90	5	0.70711	2	0.5
**German credit (GC)**	15	0.0039062	130	5	0.022097	2	0.5
**Credit card clients (CCC)**	5	0.5	130	5	0.088388	12	0.5

Supplementary Table S3. Statistical performance measures. TP and TN denote true positive and true negative predictions, respectively. FP and FN represent the false positive and false negative predictions, respectively.

Measure	Equation
Accuracy	$(TP + TN)/(TP + FN + TN + FP)$
Precision	$TP/(TP + FP)$
F1 score	$(2 \times TP)/(2 \times TP + FP + FN)$

Supplementary Table S4. Statistical measures for the 12 datasets (2/3 of data for training, 1/3 for testing). Best performing model based on F1 score is highlighted in red.

		Datasets					
		**Colon tumor**	**EEG eye state**	**Heart statlog**	**Central nervous system**	**Pima Indian diabetes**	**Synthetic**
**C4.5**	Acc	0.85000	0.55598	0.75556	0.65000	0.74902	0.93393
	Sen	0.57143	0.01071	0.86000	0.00000	0.66292	0.91892
	Spe	1.00000	1.00000	0.62500	1.00000	0.79518	0.94895
	Prec	1.00000	1.00000	0.74138	0.00000	0.63441	0.94737
	F1	0.72727	0.02119	0.79630	0.00000	0.64835	0.93293
**MLR**	Acc	0.85000	0.55678	0.85556	0.60000	0.80000	0.93093
	Sen	0.57143	0.01249	0.90000	0.14286	0.57303	0.92192
	Spe	1.00000	1.00000	0.80000	0.84615	0.92169	0.93994
	Prec	1.00000	1.00000	0.84906	0.33333	0.79688	0.93884
	F1	0.72727	0.02468	0.87379	0.20000	0.66667	0.93030
**RF**	Acc	0.85000	0.56179	0.83333	0.65000	0.76078	0.99850
	Sen	0.57143	0.02365	0.90000	0.14286	0.62921	1.00000
	Spe	1.00000	1.00000	0.75000	0.92308	0.83133	0.99700
	Prec	1.00000	1.00000	0.81818	0.50000	0.66667	0.99701
	F1	0.72727	0.04621	0.85714	0.22222	0.64740	0.99850
**ANN**	Acc	0.85000	0.55678	0.83333	0.65000	0.80000	0.98649
	Sen	0.57143	0.01249	0.84000	0.00000	0.64045	0.98499
	Spe	1.00000	1.00000	0.82500	1.00000	0.88554	0.98799
	Prec	1.00000	1.00000	0.85714	0.00000	0.75000	0.98795
	F1	0.72727	0.02468	0.84849	0.00000	0.69091	0.98647
**OmniGA**	Acc	0.95000	0.59744	0.87778	0.65000	0.79216	1.00000
	Sen	0.85714	0.13030	0.96000	0.71429	0.82023	1.00000
	Spe	1.00000	0.97783	0.77500	0.61539	0.77711	1.00000
	Prec	1.00000	0.82720	0.84211	0.50000	0.66364	1.00000
	F1	**0.92308**	**0.22514**	**0.89720**	**0.58824**	**0.73367**	**1.00000**

		**TF combinations**	**Lung tissue**	**Esophagus tissue**	**TIS prediction**	**German credit**	**Credit card clients**
**C4.5**	Acc	0.77668	0.91572	0.92857	0.89161	0.74475	0.82520
	Sen	0.60406	0.26389	0.52273	0.88594	0.81974	0.34042
	Spe	0.94175	0.98064	0.96861	0.89729	0.57000	0.96289
	Prec	0.90840	0.57576	0.62162	0.89611	0.81624	0.72265
	F1	0.72561	0.36191	0.56790	0.89100	0.81799	0.46282
**MLR**	Acc	0.80893	0.91195	0.92245	0.90986	0.79580	0.81060
	Sen	0.68020	0.30556	0.40909	0.90624	0.88841	0.21564
	Spe	0.93204	0.97234	0.97309	0.91349	0.58000	0.97958
	Prec	0.90541	0.52381	0.60000	0.91285	0.83133	0.75000
	F1	0.77681	0.38597	0.48649	0.90953	0.85892	0.33497
**RF**	Acc	0.89826	0.93459	0.94490	0.91481	0.75676	0.81960
	Sen	0.81980	0.51389	0.45455	0.91349	0.91846	0.35353
	Spe	0.97330	0.97649	0.99327	0.91614	0.38000	0.95198
	Prec	0.96707	0.68519	0.86957	0.91592	0.77536	0.67647
	F1	0.88736	0.58730	0.59702	0.91470	0.84086	0.46437
**ANN**	Acc	0.84740	0.92327	0.92857	0.91953	0.78679	0.81970
	Sen	0.79188	0.48611	0.36364	0.91542	0.88412	0.28526
	Spe	0.90049	0.96681	0.98431	0.92364	0.56000	0.97150
	Prec	0.88385	0.59322	0.69565	0.92300	0.82400	0.73974
	F1	0.83534	0.53435	0.47761	0.91919	0.85300	0.41175
**OmniGA**	Acc	0.91191	0.94088	0.94490	0.92134	0.81081	0.81260
	Sen	0.86294	0.56944	0.61364	0.91711	0.92275	0.49638
	Spe	0.95874	0.97787	0.97758	0.92557	0.55000	0.90241
	Prec	0.95238	0.71930	0.72973	0.92493	0.82692	0.59096
	F1	**0.90546**	**0.63566**	**0.66667**	**0.92101**	**0.87221**	**0.53956**

Supplementary Table S5. Statistical measures for the 12 datasets from a 3-fold crossvalidation. Best performing model based on F1 score is highlighted in red.

		Datasets					
		**Colon tumor**	**EEG eye state**	**Heart statlog**	**Central nervous system**	**Pima Indian diabetes**	**Synthetic**
**C4.5**	Acc	0.65000	0.60304	0.78889	0.65000	0.73726	0.93393
	Sen	0.42857	0.41440	0.87333	0.00000	0.64045	0.93794
	Spe	0.76923	0.75666	0.68333	1.00000	0.78916	0.92993
	Prec	0.56566	0.73460	0.77548	0.00000	0.62167	0.93078
	F1	0.45724	0.39030	0.82141	0.00000	0.63001	0.93417
**MLR**	Acc	0.80000	0.57874	0.82963	0.63333	0.76601	0.94695
	Sen	0.66667	0.50231	0.86000	0.23810	0.58052	0.94294
	Spe	0.87180	0.64099	0.79167	0.84615	0.86546	0.95095
	Prec	0.77976	0.69535	0.83818	0.46667	0.70430	0.95054
	F1	0.70274	0.41531	0.84849	0.31111	0.63479	0.94666
**RF**	Acc	0.78333	0.45617	0.82593	0.68333	0.74902	0.99900
	Sen	0.71429	0.46215	0.87333	0.28571	0.65169	1.00000
	Spe	0.82051	0.45131	0.76667	0.89744	0.80121	0.99800
	Prec	0.73333	0.58754	0.82372	0.66667	0.63992	0.99800
	F1	0.69471	0.33410	0.84760	0.37607	0.64307	0.99900
**ANN**	Acc	0.78333	0.55304	0.84074	0.65000	0.74641	0.99099
	Sen	0.66667	0.00417	0.85333	0.00000	0.63670	0.98899
	Spe	0.84615	1.00000	0.82500	1.00000	0.80522	0.99299
	Prec	0.75661	0.33333	0.85890	0.00000	0.64472	0.99300
	F1	0.68885	0.00823	0.85586	0.00000	0.63844	0.99097
**OmniGA**	Acc	0.83333	0.64964	0.84444	0.65000	0.74248	0.99950
	Sen	0.80952	0.46929	0.92000	0.57143	0.82023	0.99900
	Spe	0.84615	0.79651	0.75000	0.69231	0.70080	1.00000
	Prec	0.76852	0.71356	0.82139	0.50000	0.59856	1.00000
	F1	**0.78269**	**0.49913**	**0.86786**	**0.51337**	**0.69104**	**0.99950**

		**TF combinations**	**Lung tissue**	**Esophagus tissue**	**TIS prediction**	**German credit**	**Credit card clients**
**C4.5**	Acc	0.73118	0.92830	0.91973	0.89161	0.74374	0.81957
	Sen	0.74873	0.39352	0.53788	0.88892	0.84549	0.36543
	Spe	0.71440	0.98156	0.95740	0.89431	0.50667	0.94855
	Prec	0.75984	0.66978	0.55779	0.89373	0.80035	0.67243
	F1	0.73346	0.49335	0.54619	0.89130	0.82195	0.47242
**MLR**	Acc	0.73739	0.90943	0.91973	0.90889	0.77277	0.81030
	Sen	0.76227	0.31944	0.42424	0.90752	0.88698	0.24789
	Spe	0.71359	0.96819	0.96861	0.91026	0.50667	0.97004
	Prec	0.75192	0.51069	0.57547	0.91003	0.80766	0.70533
	F1	0.74486	0.38941	0.48743	0.90877	0.84536	0.36552
**RF**	Acc	0.80480	0.94046	0.94694	0.91179	0.76376	0.81557
	Sen	0.86548	0.50926	0.55303	0.90914	0.92275	0.38065
	Spe	0.74676	0.98340	0.98580	0.91445	0.39333	0.93909
	Prec	0.80465	0.76012	0.80429	0.91399	0.78015	0.64130
	F1	0.82177	0.60861	0.64887	0.91156	0.84538	0.47678
**ANN**	Acc	0.75682	0.93585	0.93537	0.91929	0.76577	0.81743
	Sen	0.79357	0.52778	0.44697	0.91477	0.88269	0.30530
	Spe	0.72168	0.97649	0.98356	0.92380	0.49333	0.96289
	Prec	0.75012	0.69601	0.72118	0.92324	0.80284	0.70178
	F1	0.76653	0.59937	0.54856	0.91890	0.84072	0.42458
**OmniGA**	Acc	0.80232	0.94382	0.94830	0.91985	0.78178	0.79977
	Sen	0.90102	0.55093	0.67424	0.91606	0.90987	0.51914
	Spe	0.70793	0.98294	0.97534	0.92364	0.48333	0.87947
	Prec	0.78393	0.76507	0.73335	0.92318	0.80420	0.55247
	F1	**0.82757**	**0.63988**	**0.70056**	**0.91956**	**0.85375**	**0.53442**

Supplementary Table S6. OmniGA parameters for the eleven datasets

**Dataset**	Population size	Number iterations	Prob. Crossover	Prob. Mutation	Objective function
TF combinations (TFC)	30	15	0.60	0.10	Acc
Lung tissue (LT)	30	15	0.55	0.10	F1
Esophagus tissue (ET)	30	15	0.80	0.15	F1
TIS prediction (TIS)	30	15	0.80	0.10	Acc
EEG eye state (EES)	50	25	0.85	0.05	Acc
Heart statlog (HS)	30	15	0.80	0.25	F1
Pima Indian diabetes (PID)	30	15	0.60	0.25	F1
C. nervous system (CNS)	30	15	0.85	0.05	F1
Colon tumor (CT)	30	15	0.95	0.05	F1
Synthetic (SYN)	5	5	0.85	0.05	Acc
German credit (GC)	50	25	0.85	0.05	Acc
Credit card clients	30	15	0.65	0.25	F1

Supplementary Table S7. OmniGA add-on configuration

Dataset	Early stopping	Deep learning	SMOTE
TF combinations (TFC)	False	True	False
Lung tissue (LT)	True	False	True
Esophagus tissue (ET)	False	True	True
TIS prediction (TIS)	False	False	False
EEG eye state (EES)	True	True	False
Heart statlog (HS)	True	False	False
P. Indian diabetes (PID)	False	False	True
C. nervous system (CNS)	True	False	True
Colon tumor (CT)	False	False	True
Synthetic (SYN)	False	True	True
German credit (GC)	True	False	True
Credit card clients (CCC)	True	False	True

Supplementary Table S8. OmniGA running time. Table shows the average time in seconds for each GA iteration in a 3-fold cross-validation. OmniGA programs were executed in a node with 64 cores (AMD model 6376) and CentOS 7.

Dataset	Seconds / GA iteration
TF combinations (TFC)	280
Lung tissue (LT)	200
Esophagus tissue (ET)	160
TIS prediction (TIS)	680
EEG eye state (EES)	350
Heart statlog (HS)	50
Pima Indian diabetes (PID)	75
Central nervous system (CNS)	58
Colon tumor (CT)	70
Synthetic (SYN)	84
German credit (GC)	180
Credit card clients (CCC)	1650

Supplementary Table S9. OmniGA model prediction scheme performance. Results represent the F1 score based on the validation set for a 3-fold cross-validation. Best performing prediction scheme is in bold.

Dataset	Fittest ODT model prediction (F1 score)	RF aggregation prediction (F1 score)	Voting prediction (F1 score)

TF combinations (TFC)	0.89350	0.88803	**0.89559**
Lung tissue (LT)	**0.65418**	0.63370	**0.65418**
Esophagus tissue (ET)	**0.78022**	0.72717	**0.78022**
TIS prediction (TIS)	0.91366	0.91150	**0.91689**
EEG eye state (EES)	0.72139	**0.73853**	0.72252
Heart statlog (HS)	0.87164	0.86021	**0.88080**
P. Indian diabetes (PID)	0.68009	0.63001	**0.68321**
C. nervous system (CNS)	0.66666	0.66666	**0.76666**
Colon tumor (CT)	**1.00000**	0.86666	**1.00000**
Synthetic (SYN)	**0.99668**	**0.99668**	**0.99668**
German credit (GC)	**0.87258**	0.86909	0.87201
Credit card clients (CCC)	**0.50652**	0.45402	0.47611

Supplementary Material 1

Dataset description

To evaluate the utility of the OmniGA framework, we used a set of different datasets. The first dataset is a synthetic dataset generated based on certain rules described below, four datasets constructed from real-world data, mostly from the field of genetics, four datasets from the UCI machine learning repository[1] and three from Kent Ridge Bio-medical datasets[2]. These datasets are available at www.cbrc.kaust.edu.sa/omniga/.

1. Synthetic Dataset

We used DatGen[3] program to construct a synthetic dataset with random rules. DatGen has been widely used by researchers to evaluate learning algorithms performance against well-understood synthetic datasets. The synthetic dataset contains 1000 samples in total (500 from class 1 and 500 for class 2). Each sample consists of 45 relevant features (used by the activation rules) and 20 randomly-valued features representing noise in the data. The dataset was generated using the following command:

```
./datgen -f rules.txt -r2 -
XR:R:R:R:R:R:R:R:R:R:R:R:R:R:R:R:R:R:R:R:R:R:R:R:R:R:R:R:R:R:R:R:R:R:R:R:R:R:R:R:R:R:R:R:R:I:I:I:I:I:I:
I:I:I:I:I:I:I:I:I:I:I:I:I:I -e0.10 -m0.00 -I0 -M0 -R2 -C250/350 -T150/250 -D15/20 -d500/10000 -O10000 >
features_synth.txt
```

2. Transcription factors-derived dataset

Transcription factors (TFs) are regulatory proteins that control the activation and behavior of genes in the vicinity of sites in the genomic sequence to which they bind. This dataset containing 2,364 samples derived from the known and unknown combinations of mutual binding of two TFs. The combinations of two TFs that are known to mutually bind represent positive samples, while those that are not known to mutually bind represent negative samples. Each combination (sample) is characterized by 3,186 features. Refer to Schmeier *et al.*[4] for more details.

3. Dataset based on Translation Initiation Sites surroundings from genomic sequences in *Arabidopsis Thaliana*

Translation Initiation Sites (TIS) in genomic sequences are motifs, almost always ATG nucleotide triplets, which correspond to translation start sites. The dataset contains samples defining the surroundings of a DNA sequence with the ATG at the center of the sequence with the aim to predict whether a given ATG motif in the genomic sequence of Arabidopsis is a true TIS based on properties of its surroundings. This dataset consists of 12,416 positive and 12,416 negative samples (ATG motifs that do not indicate a true TIS) encoded into vectors of 47 real-value features[5,6].

4. UCI machine learning repository datasets

We used five datasets from UCI machine learning repository[1]:

Heart statlog dataset. The dataset consists of 240 samples described by 13 features.

Pima Indians diabetes dataset. This dataset contains 8 features derived from ADAP routine for 768 females of Pima Indian heritage[7].

EEG eye state. This datasets is derived from one continuous EEG measurement with the Emotiv EEG Neuroheadset. It contains 14,980 samples and 14 features.

Credit card clients. This dataset was derived from the customers' default payments in Taiwan. It consists of 30,000 samples defined by 24 numerical attributes[8].

German credit. Dataset version produced by Strathclyde University, which converts the categorical attributes into numerical attributes. This dataset contains 1,000 samples defines by 24 attributes.

5. Kent Ridge Bio-medical datasets

We used the next three datasets from Kent Ridge Bio-medial repository:

Colon tumor dataset: Contains 62 samples collected from colon-cancer patients. Among them, 40 tumor biopsies are from tumors (labeled as "negative") and 22 normal (labeled as "positive") biopsies are from healthy parts of the colons of the same patients. Each sample is defined by 2,000 features[9].

Central nervous system dataset: Patients outcome prediction for central nervous system embryonal tumor. The dataset contains 42 patient samples, 21 are survivors (labeled as "Class1") and 39 are failures (labeled as "Class0"). Samples are defined by 7,129 numeric features (corresponding to different genes)[10].

6. FANTOM5 enhancers

Two different tissues (datasets) were considered from Kleftogiannis *et al.*[11].

Lung tissue: this datasets contains 217 samples were each corresponds to enhancers in lung tissue. Samples are defined by 351 features extracted from the sequence itself. Negative samples are random DNA sequences that are not real enhancers or promoters but mimic enhancer sequence properties. The dataset consists of 217 enhancers and 2,170 random DNA sequences.

Esophagus tissue: this datasets contains 134 samples were each corresponds to enhancers in esophagus tissue. Samples are defined by 351 features extracted from the sequence itself. Similarly to lung dataset, negative samples are random DNA sequences. The dataset consists of 134 enhancers and 1,340 random DNA sequences.

Supplementary Material 2

Data normalization and feature selection

Feature values for all datasets were normalized according to

$$\mathrm{norm_i} = \frac{\mathrm{x_i} - (\mathrm{max_i} + \mathrm{min_i})/2}{(\mathrm{max_i} - \mathrm{min_i})/2}$$

in order to have values within the range of [-1, 1], where *max* and *min* refer to the maximum and minimum values for *i*-th feature and *x* is the feature value that will be normalized. To avoid biased predictions, it is important to note that *max* and *min* values are obtained exclusively from the training data and are used as part of the model for the normalization of validation and test data.

Feature selection was performed only on datasets described by more than 300 features. We used the gain ratio method from WEKA v3.6.12[12] to evaluate and rank the features. Such a method ranks the features based on the ratio of its gain with respect to the class, divided by its entropy. We retained only the top 300 ranked features for further analyses. Similarly, for feature normalization, feature ranking is performed based exclusively on the training set. The resulting feature ranking from the training set is consequently used in the validation and test sets.

Supplementary Material 3

3.1 *Encoding models in non-terminal nodes*

OmniGA optimizes: ODT size, model selection process and split threshold, by encoding three bits of information for each non-terminal node as follows:

- Pruning (binary bit): indicates whether a non-terminal node is active (1) or pruned (0).
- Model selection (numeric bit): specifies the model for a node, where values -1, 0, 1, 2 and 3 represent leaf, ANN, C4.5, RF and MLR, respectively.
- Split threshold (numeric bit): specifies a value within range of [-0.65, 0.65] representing the threshold for splitting the data in each non-terminal node. The range [-0.65, 0.65] was chosen based on experimental results (results not shown), and allows for the more correct splitting of samples with weak class assignments (predicted scores close to 0). This threshold is applied to the predicted score of an instance (within range -1 and 1) at each non-terminal node.

The length of a GA string is determined as $\text{length} = (3 + b)\times num\_nodes$, where 3 refers to the encoded parameters used for pruning, model selection and split threshold mentioned above; b and *num_nodes* indicate the number of bits needed to encode the parameters of a model and the total number of nodes in the tree, respectively.

3.2 C4.5 model encoding

Pruning confidence factor and the stopping criteria are two important parameters for the induction of C4.5 DTs. Confidence factor is a value within the range of [0, 0.99] and sets a trade-off between tree size and accuracy, while the stopping criteria is the minimum number of samples required in a node for further expansion. Each C4.5 is encoded with 9 bits in the GA string (Supplementary Fig. 1B). These bits represent:

- Confidence factor exponential (4 binary bits): negative exponential in the binary code to calculate confidence factor = $2^{-\text{CFexp}}$ (value from 0.0078 capped to 0.99).
- Minimum number of instances (5 binary bits): encoded by binary code (value from 1 to 31). This value refers to the number of instances needed to decide whether the node is non-terminal or a leaf.

In ES add-on for C4.5, 9 bits are used to encode the minimum number of instances, resulting in a value within [0, 511] (Supplementary Fig. 1C).

3.3 MLR model encoding

MLR with a ridge estimator parameter aims to minimize a penalized residual sum of squares. Each MLR model is encoded by 5 bits denoting the negative exponential in the binary code to calculate ridge = $2^{-\text{ridgeExp}}$ (value from 4.65×10^{-10} to 1) as depicted in Supplementary Fig. 1B.
The ES add-on for MLR limits the number of iterations used during the minimization problem of the model. ES for MLR model is encoded by 4 bits of the GA string resulting in a value within the range of [1, 16] (Supplementary Fig. 1C).

3.4 RF model encoding

The number of trees and number of random features are encoded into 9 bits of the GA string, representing:

- Number of trees exponential (4 binary bits): exponential in the binary code to calculate the number of trees (value from 1 to 512).
- Number of random features (5 binary bits): number in the binary code (value from 1 to 31).

The OmniGA-ES add-on limits the depth for random tree expansion in RF. The ES add-on for RF is encoded by 4 bits of the GA string resulting in a value within range of [0, 15], where 0 means unlimited expansion (Supplementary Fig. 1C).

3.5 ANN model encoding

ANNs in non-terminal nodes are trained on the training data, and the validation set (15% of training data) is used to stop network training when error starts to increase. We used ANNs with one hidden layer as studies have shown that one hidden layer is sufficient to learn the data patterns as long as there are enough nodes in the hidden layer with sigmoid activation functions[13,14]. Each ANN is encoded by 7 bits (Supplementary Fig. 1B) as follows:

- Number of nodes (4 binary bits): number of nodes in the hidden layer in the binary code (from 1 to 15 number of nodes).
- Learning rate exponential (3 binary bits): binary bits encoding the negative exponential to calculate the learning rate (LR) as $\mathrm{LR} = 2^{-\mathrm{LRexp}}$ (from 0.0078 to 1).

In OmniGA with an ES add-on, the coefficient to calculate the number of iterations for ANN training is encoded by 4 binary bits (value within the range of [1, 16]). The actual number of iterations for ANN training is calculated by multiplying such a coefficient by 10, i.e., GA can choose to train the ANN using (10, 20, 30, …, 160) iterations (Supplementary Fig. 1C).

Finally, we implemented three different mutation strategies depending on the bit where a mutation occurs: 1) mutation in the bit encoding the model type, changes the bit to a random integer value within range of [-1, 3], 2) mutation in the threshold bit generates a random numeric value within range of [-0.65, 0.65], and 3) mutation at any other binary bit simply inverts the current value (0 → 1 or 1 → 0).

Supplementary Figures

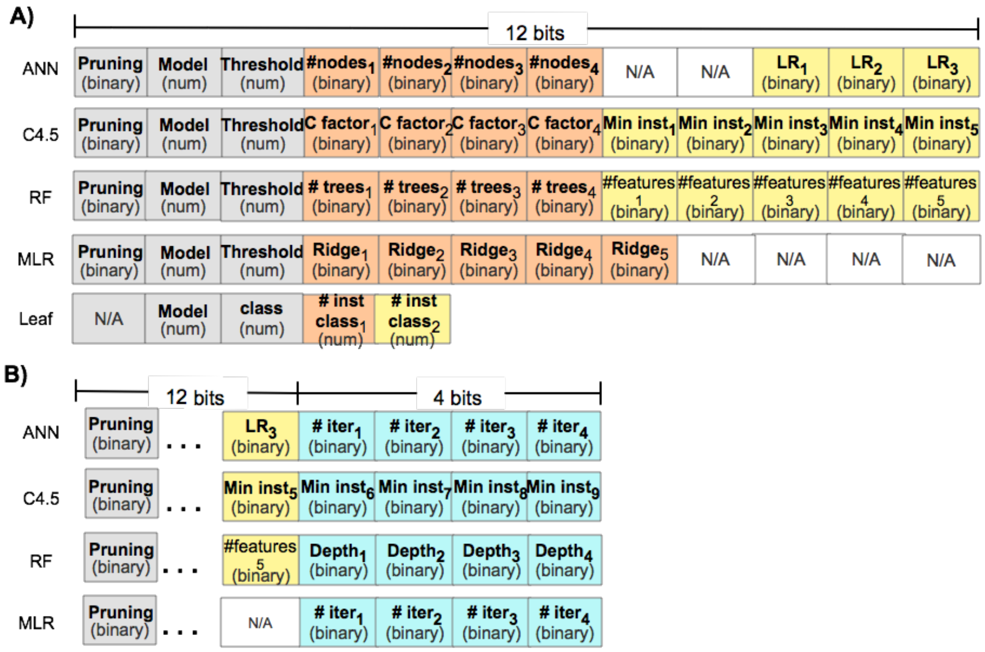

Supplementary Fig. 1. A) GA model encoding process. Each classifier is encoded by 12 bits. B) GA model encoding for OmniGA using ES add-on for model training. Four bits are added to the original encoding process to encode for ES.

References

1 Bache, K. & Lichman, M. UCI Machine Learning Repository. *Irvine, CA: University of California, School of Information and Computer Science.* (2013).

2 Li, J., Liu, H. & Wong, L. Mean-entropy discretized features are effective for classifying high-dimensional biomedical data. *The 3rd ACM SIGKDD Workshop on Data Mining in Bioinformatics*, 17-24 (2003).

3 Melli, G. The datgen Dataset Generator. *Version 3.1* (1999).

4 Schmeier, S., Jankovic, B. & Bajic, V. B. Simplified method to predict mutual interactions of human transcription factors based on their primary structure. *PLoS One* **6** (2011).

5 Magana-Mora, A. *et al.* Dragon TIS Spotter: an Arabidopsis-derived predictor of translation initiation sites in plants. *Bioinformatics* **29**, 117-118 (2013).

6 Magana-Mora, A. *et al.* in *Systemic Approaches in Bioinformatics and Computational Systems Biology: Recent Advances* (eds Paola Lecca, Dan Tulpan, & Kanagasabai Rajaraman) Ch. 5, 105-116 (IGI Global, 2011).

7 Smith, J. W., Everhart, J. E., Dickson, W. C., Knowler, W. C. & Johannes, R. S. Using the ADAP learning algorithm to forecast the onset of diabetes mellitus. *Proceedings of the Symposium on Computer Applications and Medical Care*, 261-265 (1988).

8 Yeh, I. C. & Lien, C. H. The comparisons of data mining techniques for the predictive accuracy of probability of default of credit card clients. *Expert Systems with Applications* **36**, 2473-2480 (2009).

9 Alon, U. *et al.* Broad patterns of gene expression revealed by clustering analysis of tumor and normal colon tissues probed by oligonucleotide arrays. *Proc Natl Acad Sci* **96**, 6745-6750 (1999).

10 Pomeroy, S. L. *et al.* Prediction of central nervous system embryonal tumour outcome based on gene expression. *Nature* **415**, 436-442 (2001).

11 Kleftogiannis, D., Kalnis, P. & Bajic, V. B. DEEP: a general computational framework for predicting enhancers. *Nucleic Acids Research* **43** (2015).

12 Witten, I. H. *et al.* Weka: Practical Machine Learning Tools and Techniques with Java Implementations. (1999).

13 Hornik, K., Stinchcombe, M. & White, H. Multilayer feedforward networks are universal approximators. *Neural Networks* **2**, 359-366 (1989).

14 Cybenko, G. Approximation by superpositions of a sigmoidal function. *Math. Control Signals Systems* **2**, 303-314 (1989).
